# Prevalence of depression in patients with advanced cancer receiving palliative care: a meta-analysis of self‐report instruments

**DOI:** 10.3389/fpsyt.2026.1865303

**Published:** 2026-06-24

**Authors:** Han-Yu Deng, Min Li, Xu Xu, Qian-Qian Mou

**Affiliations:** 1 Division of Pancreatic Surgery, Department of General Surgery, West China Hospital, Sichuan University, Chengdu, Sichuan, China; 2 Division of Vascular Surgery, Department of General Surgery, West China Hospital, Sichuan University, Chengdu, China; 3 Clinical Trial Center, West China Hospital, Sichuan University/West China School Of Nursing, Sichuan University, Chengdu, China

**Keywords:** advanced cancer, depression, meta-analysis, palliative care, prevalence

## Abstract

**Background:**

Although the prevalence of depression in patients with advanced cancer receiving palliative care has been widely reported, the estimates vary substantially. This meta-analysis aimed to determine the pooled prevalence of depression in patients with advanced cancer receiving palliative care and identify its potential moderating factors.

**Methods:**

A comprehensive literature search was conducted in PubMed, Web of Science, Scopus, Embase, Cochrane Library, CINAHL, and PsycINFO from inception to April 1, 2026. We calculated pooled prevalence estimates using a random-effects model and assessed heterogeneity using the I² statistic. Subgroup analyses and meta-regression were performed to explore potential sources of heterogeneity.

**Results:**

A total of 29 studies comprising 6054 patients with advanced cancer receiving palliative care were included, yielding a pooled prevalence of depression of 50.9% (95% CI: 41.2-60.5%). Subgroup analyses and meta-regression showed that the pooled prevalence varied significantly by study design and assessment tool.

**Conclusions:**

Our findings indicated that depression was very prevalent in patients with advanced cancer receiving palliative care. However, the pooled estimate should be interpreted with caution because the extremely high statistical heterogeneity remained largely unexplained, even after conducting subgroup analyses and meta-regression. Furthermore, the adoption of suitable assessment tools and the implementation of valid screening and care strategies are essential to alleviate emotional distress in this vulnerable group.

**Systematic review registration:**

PROSPERO, identifier CRD420261368352

## 1 Introduction

Cancer has become a leading cause of mortality worldwide, imposing a heavy burden on healthcare systems (1). A considerable proportion of patients are diagnosed at an advanced stage, mainly attributable to the insidious nature of cancer symptoms, the lack of effective early screening programs, and the inaccessibility of timely diagnostic services in many regions (2). When the disease cannot be cured, palliative care thus becomes the preferred option for advanced cancer patients (3). Palliative care aims to alleviate suffering through early identification and management of distressing symptoms, thereby improving patients’ quality of life (4). However, among the various distressing symptoms that undermine quality of life, depression is one of the most common and yet generally under-recognized conditions in patients with advanced cancer receiving palliative care (5).

Depression is a clinical syndrome characterized by persistent low mood and loss of interest or pleasure in activities (6). Owing to the persistent psychological stressor constituted by uncontrollable physical symptoms, functional decline, fear of death, and existential distress associated with advanced disease, depression is very common in patients with advanced cancer receiving palliative care (7, 8). Furthermore, depression has been found to be associated with increased pain, reduced treatment adherence, impaired communication with healthcare providers, and decreased overall well-being and sense of dignity in these patients (9–11). These findings highlight the need for depression screening and intervention in patients with advanced cancer in palliative care settings.

In recent years, an increasing number of standardized self-report scales have been used to assess depression in patients with advanced cancer receiving palliative care. Unlike structured clinical interviews, self-report screening tools are cost-effective, easy to administer, and suitable for large-scale surveys, making them particularly valuable for capturing patient-experienced depression in clinical settings (12). A large number of previous studies have reported the prevalence of depression in patients with advanced cancer receiving palliative care, with estimates ranging from about 10% to over 88% (13–16). This substantial variation is likely due to differences in study characteristics, including participants’ demographics, geographic region, economic status, study design, and the instruments used to assess depression. A previous meta-analysis has synthesized the prevalence of depression in cancer patients (17), but no study has specifically focused on patients with advanced cancer receiving palliative care. Moreover, given the widespread use of self-report scales in palliative care research and clinical practice, it is crucial to provide real-world evidence regarding the performance of these instruments in this vulnerable population. To address this gap, we conducted a meta-analysis with the primary aim of calculating the pooled prevalence of depression in patients with advanced cancer receiving palliative care and the secondary aim of examining moderators of the prevalence.

## 2 Methods

We conducted this meta-analysis in compliance with the Preferred Reporting Items for Systematic Reviews and Meta-Analyses (PRISMA) guidelines (18) and registered the protocol with PROSPERO (CRD420261368352).

### 2.1 Search strategy

A comprehensive search of PubMed, Web of Science, Scopus, Embase, Cochrane Library, CINAHL, and PsycINFO was carried out from the inception of each target database to April 1, 2026 (**Supplementary Table 1**). Using Boolean logic, the search strategy combined MeSH terms and corresponding free keywords, such as neoplasms, advanced cancer, palliative care, depression, and prevalence. We obtained additional eligible studies by manually checking the reference lists of all included studies.

### 2.2 Eligibility criteria

Studies were eligible if they: (1) included patients with advanced cancer (defined as unresectable, locally advanced, or metastatic cancer) receiving palliative care (≥ 18 years); (2) used a cross-sectional or longitudinal design; (3) employed a validated self-reported instrument to diagnose depression; (4) reported the prevalence of depression or provided sufficient information to calculate it. Conference abstracts, study protocols, case reports, reviews, duplicate publications, and studies not published in English were excluded.

### 2.3 Literature selection and data extraction

Two reviewers independently extracted data, and any disagreements were resolved by consulting a third reviewer. The following information was collected: first author, publication year, country, study design, sample size, mean age, proportion of female participants, cancer type, assessment tool, and depression prevalence.

### 2.4 Risk bias assessment

Using the Joanna Briggs Institute (JBI) critical appraisal checklist for prevalence studies (19), two reviewers independently assessed the risk of bias of each included study across nine domains, rating each item as “yes” (low risk), “no” (high risk), or “unclear/not applicable.” Studies were classified as low (≥ 70% “yes”), moderate (50%-69%), or high (≤ 49%) risk of bias, with any disagreements resolved through discussion with a third reviewer until consensus.

### 2.5 Statistical analysis

Statistical analyses were performed with Stata 15.0. Heterogeneity was evaluated using the I² statistic (with ≥ 50% indicating substantial heterogeneity), and a random-effects model was applied to estimate the pooled prevalence and its 95% confidence interval (CI) (20). To explore potential sources of heterogeneity, we conducted subgroup analyses by study design, economic condition, geographic region, and assessment tool. Moreover, univariate meta-regression was conducted to assess the correlation between covariates (publication year, sample size, mean age, proportion of female participants, study design, economic condition, geographic region, and assessment tool) and the pooled prevalence. We examined publication bias using funnel plots and Egger’s test, and assessed the robustness of the findings through leave-one-out sensitivity analysis. Statistical significance was set at two-sided P < 0.05.

## 3 Results

### 3.1 Literature selection and characteristics

**Figure 1:**
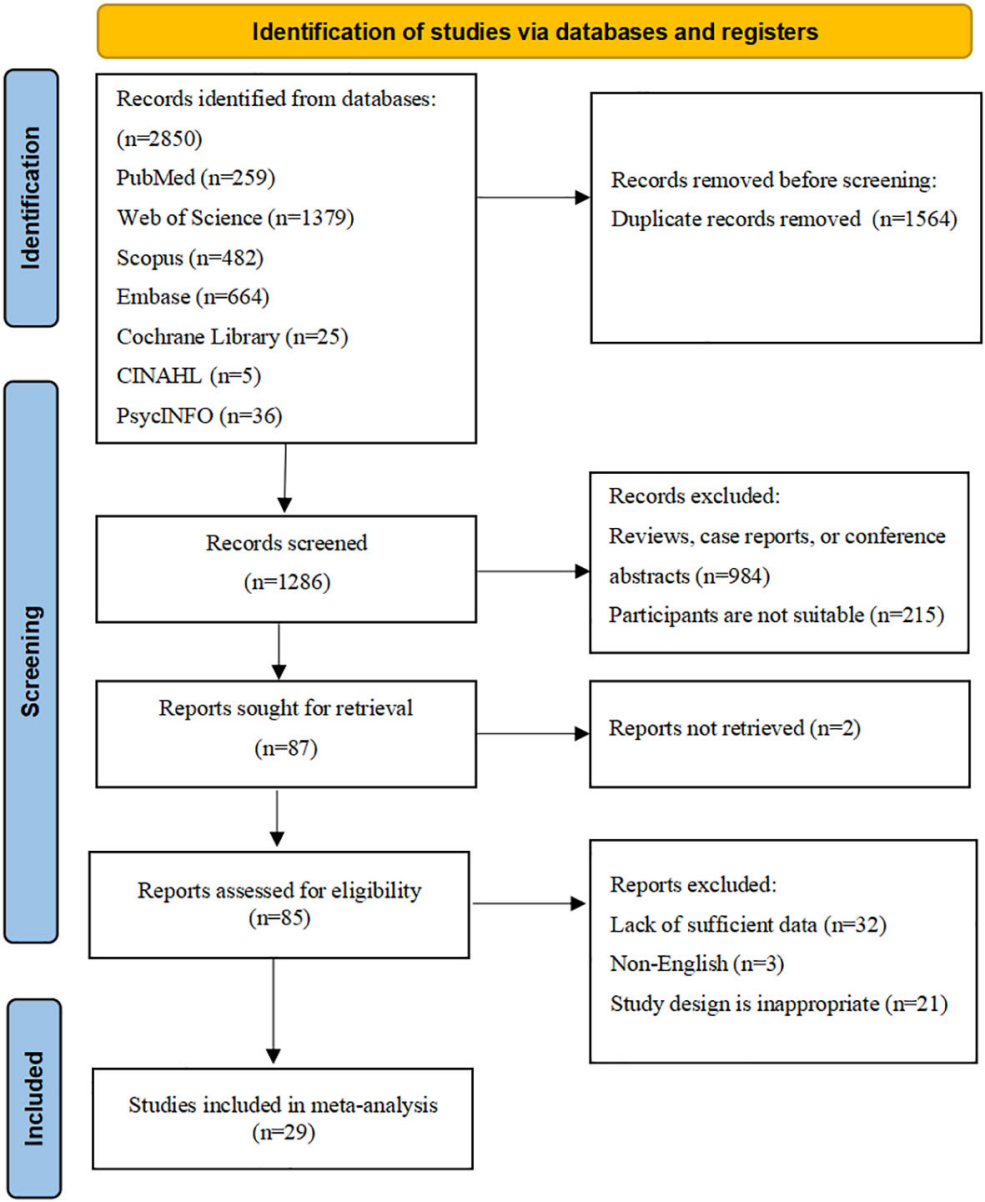
Flow diagram of study selection.

A total of 2850 records were retrieved from the initial database search. After the removal of 2821 studies owing to duplication or irrelevance, a total of 29 studies met all the inclusion criteria and were therefore included in the meta-analysis. To further address potential patient overlap across studies from the same research groups or clinical settings, we manually compared author names, institutional affiliations, study periods, geographic locations, and key sample characteristics. No overlapping cohorts were identified among the 29 included studies. The study selection process is summarized in **Figure 1**. These studies involved 6054 patients with advanced cancer receiving palliative care across 20 countries. Most of the studies employed a cross-sectional design and were conducted in high income countries. The sample sizes of individual studies ranged from 53 to 820. The majority of the studies used the Hospital Anxiety and Depression Scale-Depression subscale (HADS-D) to assess depression. Detailed characteristics of the included studies are presented in **Table 1**.

**Table 1:** Characteristics of included studies.

Study	Country	Study design	Sample size	Mean age (years)	Female (%)	Cancer type	Assessment tool	Prevalence (%)
Alotaibi and Alsuhail. 2025 (13)	Saudi Arabia	Cross-sectional	130	NA	66.9	Mixed cancer types	HADS-D	50.0
Amano et al. (2024) (14)	Japan	Cross-sectional	225	62	47.7	Mixed cancer types	PHQ-9	34.2
Atinafu et al. (2022) (21)	Ethiopia	Cross-sectional	171	47.0 ± 13.6	68.4	Mixed cancer types	HADS-D	47.4
Bovero et al. (2019) (22)	Italy	Cross-sectional	152	74.8 ± 11.4	46.7	Mixed cancer types	HADS-D	86.8
Bovero et al. (2021) (23)	Italy	Cross-sectional	350	69.0 ± 12.6	48.3	Mixed cancer types	PHQ-9	74.1
Bovero et al. (2023) (24)	Italy	Cross-sectional	170	68.8 ± 13.1	36.1	Mixed cancer types	PHQ-9	36.5
Bužgová et al. (2015) (25)	Czech Republic	Longitudinal	225	65.1 ± 12.6	49.3	Mixed cancer types	HADS-D	42.9
Chan et al. (2012) (26)	China	Cross-sectional	53	62.1 ± 15.5	100	Gynecological cancer	HADS-D	62.3
Delgado-Guay et al. (2009) (27)	USA	Cross-sectional	216	59	40.1	Mixed cancer types	HADS-D	36.6
Gontijo Garcia et al. (2023) (28)	Brazil	Cross-sectional	70	66.1 ± 11.7	60	Mixed cancer types	HADS-D	44.3
Grotmol et al. (2017) (29)	Norway	Cross-sectional	563	63	44.0	Mixed cancer types	PHQ-9	11
Islam et al. (2022) (30)	Bangladesh	Cross-sectional	95	48.9 ± 9.9	100	Breast cancer	HADS-D	48.4
Jung et al. (2025) (31)	South Korea	Cross-sectional	144	60.7 ± 7.2	42.4	Mixed cancer types	PHQ-9	31.3
Lloyd-Williams et al. (2014) (32)	UK	Longitudinal	629	66.0 ± 12.9	67.1	Mixed cancer types	PHQ-9	31.6
Mercadante et al. (2015) (33)	Italy	Cross-sectional	820	69.7 ± 12.7	47.7	Mixed cancer types	HADS-D	48.7
Mercadante et al. (2017) (34)	Italy	Cross-sectional	219	65.4 ± 12.4	49.3	Mixed cancer types	HADS-D	85.4
Mercadante et al. (2021) (35)	Italy	Cross-sectional	182	69.9 ± 10.8	33.5	Lung cancer	HADS-D	62.6
Mystakidou et al. (2009) (36)	Greece	Cross-sectional	82	62.7 ± 13.7	56.1	Mixed cancer types	BDI	80.5
Mystakidou et al. (2012) (37)	Greece	Cross-sectional	92	73.3 ± 5.9	42.4	Mixed cancer types	GDS-15	67.4
O’Connor et al. (2010) (38)	Australia	Cross-sectional	266	70.7	45.5	Mixed cancer types	HADS-D	45.8
Park et al. (2018) (15)	South Korea	Cross-sectional	100	NA	19	Mixed cancer types	HADS-D	88
Pérez-Cruz et al. (2019) (39)	Chile	Cross-sectional	208	64.0 ± 14.0	50	Mixed cancer types	HADS-D	37.5
Rojas-Concha et al. (2023) (16)	Denmark	Longitudinal	201	66	48.3	Mixed cancer types	HADS-D	10
Sela. (2007) (40)	Canada	Cross-sectional	132	NA	52.3	Mixed cancer types	SDS	72
Sewtz et al. (2021) (41)	Germany	Longitudinal	102	68.6 ± 11.7	45.2	Mixed cancer types	HADS-D	56.7
Slovacek et al. (2009) (42)	Czech Republic	Cross-sectional	64	60.5	100	Mixed cancer types	SDS	71.8
Smith et al. (2003) (43)	UK	Cross-sectional	68	NA	51.5	Mixed cancer types	HADS-D	22.1
Sudarisan et al. (2019) (44)	India	Cross-sectional	234	67.4 ± 10.8	59.8	Mixed cancer types	PHQ-9	69.7
Truong et al. (2024) (45)	Vietnam	Cross-sectional	91	57.7 ± 12.3	53.8	Mixed cancer types	HADS-D	40

NA, Not available; HADS-D, Hospital Anxiety and Depression Scale-Depression subscale; PHQ-9, Patient Health Questionnaire-9; BDI, Beck Depression Inventory.

### GDS-15: geriatric depression scale-15; SDS: self-rating depression scale 3.2 risk bias assessment results

According to the Joanna Briggs Institute (JBI) critical appraisal checklist, 12 studies (41.4%) were assessed as having a low risk of bias, whereas the remaining 17 (58.6%) had a moderate risk of bias. In terms of the individual domains, the sample frame was appropriate in 28 studies (96.6%), the sampling method had high bias risk in all 29 (100%) due to convenience sampling, sample size was adequate in only 9 (31.0%), and unclear in 20 (69.0%). All 29 studies satisfied in description, data coverage, valid methods, reliable measurement, and appropriate analysis. For response rate, 12 (41.4%) were adequate, 4 (13.8%) inadequate, and 13 (44.8%) unclear.

### 3.3 Pooled prevalence of depression

**Figure 2:**
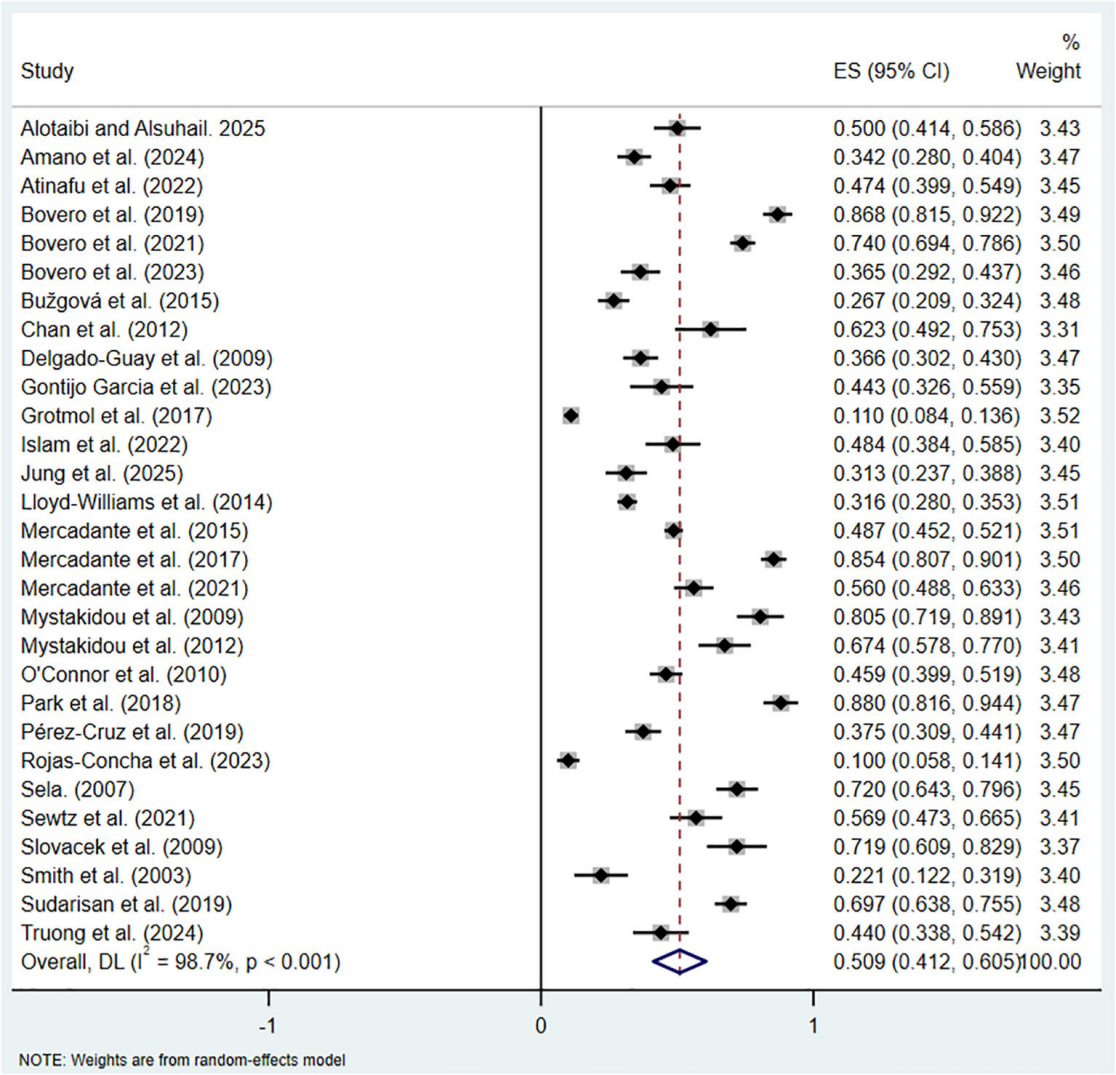
Forest plot of pooled prevalence of depression in patients with advanced cancer receiving palliative care.

Given the substantial heterogeneity (I² = 98.7%) observed among studies, we employed a random-effects model and estimated the pooled prevalence of depression in patients with advanced cancer receiving palliative care to be 50.9% (95% CI: 41.2-60.5%) (**Figure 2**).

### 3.4 Subgroup analysis and meta-regression analysis

The detailed results of the subgroup analyses are summarized in **Table 2**. As shown in **Supplementary Figure 1**, the estimated prevalence of depression in patients with advanced cancer receiving palliative care differed significantly between cross-sectional studies (54.1%, 95% CI: 43.4-64.7%) and longitudinal studies (30.8%, 95% CI: 15.6-46.0%). Moreover, the pooled prevalence of depression in high-income countries was not observed to be significantly different from that in low-middle income countries (**Supplementary Figure 2**). When analyzed by geographic region (**Supplementary Figure 3**), the pooled prevalence of depression was 52.6% (95% CI: 39.3-66.0%) in the Asia-Pacific region, 47.4% (95% CI: 39.9-54.9%) in Africa, 51.0% (95% CI: 36.0-65.9%) in Europe, and 47.6% (95% CI: 30.5-64.6%) in the Americas, with no significant difference among these groups. Subgroup analysis by assessment tool revealed a pooled prevalence of 49.8% (95% CI: 38.0-61.6%) for HADS-D, 41.2% (95% CI: 21.6-60.7%) for PHQ-9, and 73.2% (95% CI: 67.7-78.6%) for other scales, with a significant difference between subgroups (P < 0.001) (**Supplementary Figure 4**).

**Table 2:** Subgroup analyses of the pooled prevalence of depression in patients with advanced cancer receiving palliative care.

Subgroups	Number of studies	Prevalence (95% CI)	Heterogeneity		P values across subgroups
			I²	P values	
Study design					0.014
Cross-sectional	25	54.1% (43.4-64.7%)	98.7%	P < 0.001	
Longitudinal	4	30.8% (15.6-46.0%)	97.2%	P < 0.001	
Economic condition					0.754
High income	23	50.4% (39.2-61.6%)	99.0%	P < 0.001	
Low-middle income	6	52.8% (42.6-63.1%)	86.9%	P < 0.001	
Geographic region					0.906*
Asia-Pacific	9	52.6% (39.3-66.0%)	96.4%	P < 0.001	
Africa	1	47.4% (39.9-54.9%)	-	P < 0.001	
Europe	15	51.0% (36.0-65.9%)	99.2%	P < 0.001	
Americas	4	47.6% (30.5-64.6%)	94.9%	P < 0.001	
Assessment tool					< 0.001
HADS-D	18	49.8% (38.0-61.6%)	98.4%	P < 0.001	
PHQ-9	7	41.2% (21.6-60.7%)	98.2%	P < 0.001	
Other scale	4	73.2% (67.7-78.6%)	30.9%	P < 0.001	

* In the geographic region subgroup, only one study was included from Africa. Therefore, the comparison involving this subgroup has limited statistical meaning and should be interpreted with caution.

Univariate meta-regression analysis (**Table 3**) demonstrated that study design and assessment tool were significantly associated with the pooled prevalence of depression in patients with advanced cancer receiving palliative care (P < 0.05). In contrast, none of the other moderators examined, including publication year, sample size, mean age, proportion of female participants, economic condition, and geographic region, were significantly associated (all P > 0.05).

**Table 3:** Meta-regression results of the pooled prevalence of depression in patients with advanced cancer receiving palliative care.

Variables	Number of studies	Coefficient	Standard error	95%CI	T values	P values
Publication year	29	-0.0054	0.0069	(-0.0195, 0.0088)	-0.78	0.443
Sample size	29	-0.0004	0.0002	(-0.0008, 0.0001)	-1.54	0.135
Mean age	29	0.0077	0.0067	(-0.0060, 0.0215)	1.16	0.257
Proportion of female participants	29	0.0006	0.0023	(-0.0041, 0.0053)	0.28	0.784
Study design (Ref: Cross-sectional)						
Longitudinal	4	-0.2306	0.1120	(-0.4604, -0.0007)	-2.06	0.049
Economic condition (Ref: High income)						
Low-middle income	6	0.0232	0.1038	(-0.1898, 0.2363)	0.22	0.824
Geographic region (Ref: Asia-Pacific)						
Africa	1	-0.526	0.2455	(-0.5583, 0.4531)	-0.21	0.832*
Europe	15	-0.0506	0.1404	(-0.3397, 0.2385)	-0.36	0.722
Americas	4	-0.0172	0.0983	(-0.2198, 0.1853)	-0.18	0.862
Assessment tool (Ref: HADS-D)						
PHQ-9	7	-0.8683	0.1152	(-0.0056, 0.4679)	2.01	0.055
Other scale	4	0.2558	0.0412	(0.2602, 0.4856)	2.28	0.030

* In the geographic region subgroup, only one study was included from Africa. Therefore, the comparison involving this subgroup has limited statistical meaning and should be interpreted with caution.

### 3.5 Publication bias and sensitivity analysis

**Figure 3:**
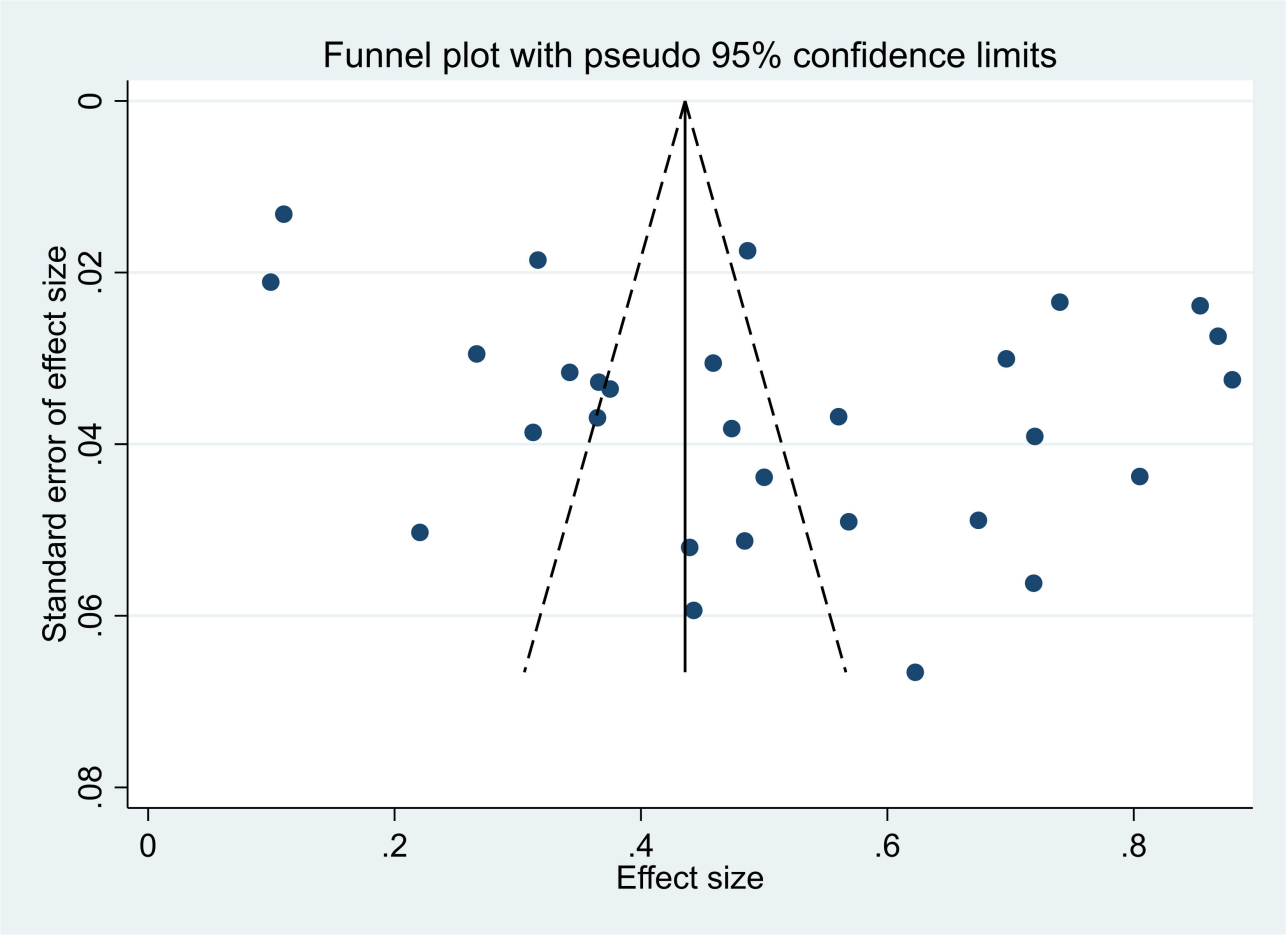
Funnel plot.

Funnel plot inspection (**Figure 3**) and Egger’s regression test indicated no significant publication bias (t = 1.35, P = 0.189; **Supplementary Figure 5**), and sensitivity analysis confirmed the robustness of the findings (**Supplementary Table 3**).

## 4 Discussion

To our knowledge, this is the first meta-analysis using self-report instruments to determine the prevalence of depression in patients with advanced cancer receiving palliative care. A pooled analysis of the 29 included studies showed that the overall prevalence of depression was 50.9% in the 6054 patients with advanced cancer receiving palliative care. The considerable burden of depression observed in patients with advanced cancer within palliative care settings is significantly greater than that documented in an earlier meta-analysis of depression among cancer patients treated in conventional clinical environments (17). This discrepancy could be attributed to a variety of physiological and psychosocial factors. First, patients with advanced cancer receiving palliative care generally have a shorter life expectancy and experience a heavier burden of physical symptoms, including pain, fatigue, dyspnea, and anorexia (25). These distressing symptoms are regarded as the significant risk factors for depression. Furthermore, with regard to psychological status, patients with advanced cancer in palliative care often face existential distress, such as fear of death, loss of dignity, and hopelessness (27). In contrast, general cancer patients may still be receiving curative or life-prolonging treatments, and such distress is less prominent. Third, patients with advanced cancer in palliative care experience severe functional decline and rely on caregivers for activities of daily living, which may undermine self-efficacy and trigger feelings of worthlessness and depression (31). Finally, patients admitted to palliative care tend to be those with higher levels of psychological distress or complex symptom needs (33), and this selection bias may lead to an observed prevalence of depression that is higher than that among unselected cancer patients in general clinical settings. Therefore, routine screening for depression in patients with advanced cancer during palliative care, along with encouraging patients to actively report their emotional status and providing timely targeted interventions, is recommended to reduce emotional distress and improve their quality of life.

It is noteworthy that the assessment tool was observed to be significantly associated with depression in patients with advanced cancer receiving palliative care. Specifically, compared with the HADS-D (49.8%), the prevalence of depression screened by the PHQ-9 (41.2%) showed no significant difference, whereas the other scales (73.2%) showed significant differences. These findings were consistent with a previous systematic review (12). However, although the HADS-D is currently the most widely used scale for depression screening in cancer patients and has acceptable screening performance, it performs relatively poorly in advanced cancer, palliative care, and the diagnosis of mild depression due to its insufficient consideration of somatic symptoms (46). Given that somatic symptoms are often core features of depression, neglecting them makes depression more difficult to identify. Moreover, although the PHQ-9 performs well in detecting depression in cancer patients (47), the pooled prevalence based on this scale was lower than that based on other tools in our analysis, possibly due to differences in measurement properties and the lack of validation specifically in advanced palliative care populations. In contrast, the higher pooled prevalence observed with other scales (BDI, CES-D, SDS) could be attributed to their inclusion of numerous somatic symptom items (48). Patients with advanced cancer receiving palliative care generally experience severe physical symptoms such as fatigue, pain, anorexia, and insomnia. When these physical complaints are counted toward depression scores without adjustment, they are likely to be overattributed to depressive disorder, leading to an inflation of the estimated prevalence. Therefore, when assessing depression in patients with advanced cancer receiving palliative care, scales containing either too many or too few somatic symptom items should be avoided, as their use may lead to overestimation or underestimation of the prevalence. Furthermore, developing a self-assessment tool for depression specifically tailored to patients with advanced cancer receiving palliative care should become a priority for future research.

Subgroup analysis and meta-regression revealed that longitudinal studies yielded a significantly lower baseline prevalence estimate (30.8%) compared with cross-sectional studies (54.1%). Although both designs assessed depression at a single time point, longitudinal studies typically have stricter inclusion criteria, excluding patients with short life expectancy, poor performance status, or cognitive impairment. These patients are the populations at highest risk for depression. Therefore, the baseline samples in longitudinal studies are systematically less depressed, leading to an underestimate relative to more inclusive cross-sectional studies. However, this comparison should be interpreted with caution given the marked imbalance in the number of studies (only 4 longitudinal studies), as pooled estimates from a small number of studies are statistically less stable and more susceptible to the influence of individual studies or publication bias. The predominance of cross-sectional designs in our meta-analysis suggested that the pooled prevalence of 50.9% may overestimate the true burden. In addition, no significant association was observed between economic condition or geographic region and the prevalence of depression in patients with advanced cancer receiving palliative care. These findings suggest that depression may be universally prevalent across economic levels and cultures among this population, but cautious interpretation is warranted given the substantial heterogeneity in assessment tools, study designs, and cultural contexts across the included studies.

## 5 Limitations

Several limitations should be acknowledged. First, while this study revealed no significant evidence of publication bias, restricting inclusion to English-language articles and excluding grey literature may have introduced such bias. Second, all included studies employed convenience sampling, meaning that the participants were not randomly selected from the target population but were recruited based on accessibility. This may over-represent patients who are more accessible, healthier, or more willing to participate, potentially leading to either underestimation or overestimation of depression prevalence. Moreover, the lack of probability sampling limits the generalizability of our findings to different healthcare settings, geographic regions, or cultural backgrounds. Notably, 69.0% of the included studies did not report whether the sample size was adequate, which further affects the precision and statistical power of the estimates. Third, although subgroup analyses and meta-regression did not indicate that economic status moderated depression prevalence, most of the included studies were conducted in high-income countries, which may limit the generalizability of our findings. Therefore, further research is warranted given the paucity of studies from low- and middle-income countries. Fourth, all included studies relied on self-report measures. While such instruments are practical and widely used in palliative care research, they are subject to inherent biases, including recall bias and social desirability bias, such that the true prevalence of depression may be over- or underestimated. Finally, in line with other prevalence meta-analyses, the included studies showed substantial heterogeneity that could only be partially accounted for by study design and assessment tool. Other potential sources, such as differences in patient characteristics (disease stage, comorbidity), variations in assessment threshold or timing, and unmeasured contextual factors, may have contributed to the remaining heterogeneity, which thus remained largely unexplained. These indicate that the pooled estimate is subject to considerable uncertainty and should be used with caution in clinical practice.

## 6 Conclusion

Our findings demonstrated that over half of patients with advanced cancer undergoing palliative care are affected by depression. However, the pooled estimate was accompanied by extremely high statistical heterogeneity that remained largely unexplained despite subgroup analyses and meta-regression accounting for study design and assessment tool. Therefore, this prevalence estimate should be interpreted with caution in clinical practice. Furthermore, given the considerable burden of depression in this vulnerable population, healthcare professionals should adopt suitable assessment instruments and implement valid depression screening and care strategies to alleviate emotional distress among patients with advanced cancer undergoing palliative care.

## Data Availability

The original contributions presented in the study are included in the article/**Supplementary Material**. Further inquiries can be directed to the corresponding author.

## References

[1] Wu Z Xia F Lin R . Global burden of cancer and associated risk factors in 204 countries and territories, 1980-2021: a systematic analysis for the GBD 2021. J Hematol Oncol. (2024) 17:119. doi: 10.1186/s13045-024-01640-8 39614359 PMC11607901

[2] Bigi S Ganfi V Borelli E Potenza L Artioli F Eliardo S . Perceptions of death among patients with advanced cancer receiving early palliative care and their caregivers: Results from a mixed-method analysis. Oncologist. (2023) 28:e54–62. doi: 10.1093/oncolo/oyac227 36320128 PMC9847550

[3] Choi YY Hong B Rha SY Cho S Lee HS Lee J . The effect of nurse-led enhanced supportive care as an early primary palliative care approach for patients with advanced cancer: A randomized controlled trial. Int J Nurs Stud. (2025) 168:105102. doi: 10.1016/j.ijnurstu.2025.105102 40378811

[4] Wang M Ding X . Integrated palliative care improves the quality of life of advanced cancer patients. BMC Palliative Care. (2025) 24:162. doi: 10.1186/s12904-025-01800-8 40483417 PMC12144830

[5] Price A Hotopf M . The treatment of depression in patients with advanced cancer undergoing palliative care. Curr Opin Supportive Palliative Care. (2009) 3:61–6. doi: 10.1097/SPC.0b013e328325d17a 19365163

[6] Yang Y Zhao X Cui M Wang Y . Dimensions of spiritual well-being in relation to physical and psychological symptoms: a cross-sectional study of advanced cancer patients admitted to a palliative care unit. BMC Palliative Care. (2023) 22:137. doi: 10.1186/s12904-023-01261-x 37710223 PMC10500771

[7] Rodríguez-Mayoral O Peña-Nieves A Allende-Pérez S Lloyd-Williams M . Comparing the hospital anxiety and depression scale to the Brief Edinburgh Depression Scale for identifying cases of major depressive disorder in advanced cancer palliative patients. Palliative Supportive Care. (2021) 19:170–4. doi: 10.1017/S1478951520000760 32830630

[8] Lie HC Hjermstad MJ Fayers P Finset A Kaasa S Loge JH . Depression in advanced cancer--assessment challenges and associations with disease load. J Affect Disord. (2015) 173:176–84. doi: 10.1016/j.jad.2014.11.006 25462414

[9] Aaron RV Ravyts SG Carnahan ND Bhattiprolu K Harte N McCaulley CC . Prevalence of depression and anxiety among adults with chronic pain: a systematic review and meta-analysis. JAMA Netw Open. (2025) 8:e250268. doi: 10.1001/jamanetworkopen.2025.0268 40053352 PMC11889470

[10] Grenard JL Munjas BA Adams JL Suttorp M Maglione M McGlynn EA . Depression and medication adherence in the treatment of chronic diseases in the United States: a meta-analysis. J Gen Internal Med. (2011) 26:1175–82. doi: 10.1007/s11606-011-1704-y 21533823 PMC3181287

[11] Akiba CF Zimba CC Thom A Matewere M Go V Pence B . The role of patient-provider communication: a qualitative study of patient attitudes regarding co-occurring depression and chronic diseases in Malawi. BMC Psychiatry. (2020) 20:243. doi: 10.1186/s12888-020-02657-2 32429877 PMC7236218

[12] Wakefield CE Butow PN Aaronson NA Hack TF Hulbert-Williams NJ Jacobsen PB . Patient-reported depression measures in cancer: a meta-review. Lancet Psychiatry. (2015) 2:635–47. doi: 10.1016/S2215-0366(15)00168-6 26303561

[13] Alotaibi NA Alsuhail AI . Prevalence of depression and anxiety in cancer patients receiving palliative care in the Comprehensive Cancer Center, King Fahad Medical City, Riyadh, Saudi Arabia: a cross-sectional study. Cureus. (2025) 17:e83493. doi: 10.7759/cureus.83493 40470430 PMC12135104

[14] Amano K Okamura S Matsuda Y Baracos VE Mori N Miura T . Associations of nutrition impact symptoms with depression in patients with advanced cancer. Supportive Care Cancer: Off J Multinational Assoc Supportive Care Cancer. (2024) 32:445. doi: 10.1007/s00520-024-08645-6 38896133

[15] Park YY Jeong YJ Lee J Moon N Bang I Kim H . The influence of family adaptability and cohesion on anxiety and depression of terminally ill cancer patients. Supportive Care Cancer: Off J Multinational Assoc Supportive Care Cancer. (2018) 26:313–21. doi: 10.1007/s00520-017-3912-4 28975413

[16] Rojas-Concha L Hansen MB Petersen MA Groenvold M . Symptoms of advanced cancer in palliative medicine: a longitudinal study. BMJ Supportive Palliative Care. (2023) 13:e415–27. doi: 10.1136/bmjspcare-2021-002999 34162585

[17] Krebber AM Buffart LM Kleijn G Riepma IC de Bree R Leemans CR . Prevalence of depression in cancer patients: a meta-analysis of diagnostic interviews and self-report instruments. Psycho-Oncology. (2014) 23:121–30. doi: 10.1002/pon.3409 24105788 PMC4282549

[18] Page MJ McKenzie JE Bossuyt PM Boutron I Hoffmann TC Mulrow CD . The PRISMA 2020 statement: an updated guideline for reporting systematic reviews. BMJ (Clinical Res Ed). (2021) 372:n71. doi: 10.1136/bmj.n71 33782057 PMC8005924

[19] Munn Z Moola S Lisy K Riitano D Tufanaru C . Methodological guidance for systematic reviews of observational epidemiological studies reporting prevalence and cumulative incidence data. Int J Evidence-Based Healthc. (2015) 13:147–53. doi: 10.1097/XEB.0000000000000054 26317388

[20] Higgins JP Thompson SG Deeks JJ Altman DG . Measuring inconsistency in meta-analyses. BMJ (Clinical Res Ed). (2003) 327:557–60. doi: 10.1136/bmj.327.7414.557 12958120 PMC192859

[21] Atinafu BT Demlew TM Tarekegn FN . Magnitude of anxiety and depression and associated factors among palliative care patients with cancer at Tikur Anbessa Specialized Hospital, Ethiopia. Ethiopian J Health Sci. (2022) 32:331–42. doi: 10.4314/ejhs.v32i2.14 35693579 PMC9175223

[22] Bovero A Botto R Adriano B Opezzo M Tesio V Torta R . Exploring demoralization in end-of-life cancer patients: prevalence, latent dimensions, and associations with other psychosocial variables. Palliative Supportive Care. (2019) 17:596–603. doi: 10.1017/S1478951519000191 31196235

[23] Bovero A Opezzo M Botto R Gottardo F Torta R . Hope in end-of-life cancer patients: a cross-sectional analysis. Palliative Supportive Care. (2021) 19:563–9. doi: 10.1017/S1478951520001388 33431082

[24] Bovero A Opezzo M Tesio V . Relationship between demoralization and quality of life in end-of-life cancer patients. Psycho-Oncology. (2023) 32:429–37. doi: 10.1002/pon.6095 36604571

[25] Bužgová R Jarošová D Hajnová E . Assessing anxiety and depression with respect to the quality of life in cancer inpatients receiving palliative care. Eur J Oncol Nurs: Off J Eur Oncol Nurs Soc. (2015) 19:667–72. doi: 10.1016/j.ejon.2015.04.006 26009311

[26] Chan KY Chan ML Yau TC Li CW Cheng HW Sham MK . Quality of life for Hong Kong Chinese patients with advanced gynecological cancers in the palliative phase of care: a cross-sectional study. J Palliative Care. (2012) 28:259–66. doi: 10.1177/082585971202800404 23413761

[27] Delgado-Guay M Parsons HA Li Z Palmer JL Bruera E . Symptom distress in advanced cancer patients with anxiety and depression in the palliative care setting. Supportive Care Cancer: Off J Multinational Assoc Supportive Care Cancer. (2009) 17:573–9. doi: 10.1007/s00520-008-0529-7 19005686

[28] Gontijo Garcia GS Meira KC de Souza AH Guimarães NS . Anxiety and depression disorders in oncological patients under palliative care at a hospital service: a cross-sectional study. BMC Palliative Care. (2023) 22:116. doi: 10.1186/s12904-023-01233-1 37580770 PMC10426043

[29] Grotmol KS Lie HC Hjermstad MJ Aass N Currow D Kaasa S . Depression-a major contributor to poor quality of life in patients with advanced cancer. J Pain Symptom Manage. (2017) 54:889–97. doi: 10.1016/j.jpainsymman.2017.04.010 28803091

[30] Islam N Biswas J Kowshik MM Molla MMA Saker M Chowdhury MK . Depression, anxiety, and performance status among the women with metastatic breast cancer receiving palliative care in Bangladesh: a cross sectional study. Health Sci Rep. (2022) 5:e911. doi: 10.1002/hsr2.911 36320658 PMC9617648

[31] Jung JY Yun JY Kang JH Koh SJ Kim YJ Seo S . Interaction effect of comorbid depression and proactive positivity coping strategy on the 1-year survival of patients with advanced cancer: a nationwide multicentre study in South Korea. BMC Psychiatry. (2025) 25:565. doi: 10.1186/s12888-025-06972-4 40457290 PMC12131379

[32] Lloyd-Williams M Payne S Reeve J Dona RK . Thoughts of self-harm and depression as prognostic factors in palliative care patients. J Affect Disord. (2014) 166:324–9. doi: 10.1016/j.jad.2014.05.029 25012448

[33] Mercadante S Aielli F Adile C Ferrera P Valle A Cartoni C . Sleep disturbances in patients with advanced cancer in different palliative care settings. J Pain Symptom Manage. (2015) 50:786–93. doi: 10.1016/j.jpainsymman.2015.06.018 26311122

[34] Mercadante S Adile C Ferrera P Masedu F Valenti M Aielli F . Sleep disturbances in advanced cancer patients admitted to a supportive/palliative care unit. Supportive Care Cancer: Off J Multinational Assoc Supportive Care Cancer. (2017) 25:1301–6. doi: 10.1007/s00520-016-3524-4 27957622

[35] Mercadante S Valle A Cartoni C Pizzuto M . Insomnia in patients with advanced lung cancer admitted to palliative care services. Int J Clin Pract. (2021) 75:e14521. doi: 10.1111/ijcp.14521 34120396

[36] Mystakidou K Parpa E Tsilika E Gennatas C Galanos A Vlahos L . How is sleep quality affected by the psychological and symptom distress of advanced cancer patients? Palliative Med. (2009) 23:46–53. doi: 10.1177/0269216308098088 18838488

[37] Mystakidou K Parpa E Tsilika E Panagiotou I Zygogianni A Giannikaki E . Geriatric depression in advanced cancer patients: the effect of cognitive and physical functioning. Geriatr Gerontol Int. (2013) 13:281–8. doi: 10.1111/j.1447-0594.2012.00891.x 22694340

[38] O'Connor M White K Kristjanson LJ Cousins K Wilkes L . The prevalence of anxiety and depression in palliative care patients with cancer in Western Australia and New South Wales. Med J Aust. (2010) 193:S44–7. doi: 10.5694/j.1326-5377.2010.tb03927.x 21542445

[39] Pérez-Cruz PE Langer P Carrasco C Bonati P Batic B Tupper Satt L . Spiritual pain is associated with decreased quality of life in advanced cancer patients in palliative care: an exploratory study. J Palliative Med. (2019) 22:663–9. doi: 10.1089/jpm.2018.0340 30649985

[40] Sela RA . Screening for depression in palliative cancer patients attending a pain and symptom control clinic. Palliative Supportive Care. (2007) 5:207–17. doi: 10.1017/s1478951507000375 17969824

[41] Sewtz C Muscheites W Grosse-Thie C Kriesen U Leithaeuser M Glaeser D . Longitudinal observation of anxiety and depression among palliative care cancer patients. Ann Palliative Med. (2021) 10:3836–46. doi: 10.21037/apm-20-1346 33832298

[42] Slovacek L Slovackova B Slanska I Priester P Petera J Kopecky J . Cancer and depression: a prospective study. Neoplasma. (2009) 56:187–93. doi: 10.4149/neo_2009_03_187 19309220

[43] Smith EM Gomm SA Dickens CM . Assessing the independent contribution to quality of life from anxiety and depression in patients with advanced cancer. Palliative Med. (2003) 17:509–13. doi: 10.1191/0269216303pm781oa 14526884

[44] Sudarisan SSP Abraham B George C . Prevalence, correlates of depression, and its impact on quality of life of cancer patients attending a palliative care setting in South India. Psycho-Oncology. (2019) 28:1308–13. doi: 10.1002/pon.5083 30950122

[45] Truong QXN Than TNH Le Dai D Duong KD Krakauer EL Herman B . Inclusion of social work in comprehensive palliative care to address psychosocial needs of advanced cancer patients in Vietnam. J Soc Work End-of-Life Palliative Care. (2024) 20:147–60. doi: 10.1080/15524256.2024.2310863 38346173

[46] Mitchell AJ Meader N Symonds P . Diagnostic validity of the Hospital Anxiety and Depression Scale (HADS) in cancer and palliative settings: a meta-analysis. J Affect Disord. (2010) 126:335–48. doi: 10.1016/j.jad.2010.01.067 20207007

[47] Hinz A Mehnert A Kocalevent RD Brähler E Forkmann T Singer S . Assessment of depression severity with the PHQ-9 in cancer patients and in the general population. BMC Psychiatry. (2016) 16:22. doi: 10.1186/s12888-016-0728-6 26831145 PMC4736493

[48] Mitchell AJ . Short screening tools for cancer-related distress: a review and diagnostic validity meta-analysis. J Natl Compr Cancer Netw: JNCCN. (2010) 8:487–94. doi: 10.6004/jnccn.2010.0035 20410338

