# Supplementary material for: Prevalence of depression in patients with advanced cancer receiving palliative care: a meta-analysis of self‐report instruments
Source: Front Psychiatry. 2026 Jun 24;17:1865303. doi: 10.3389/fpsyt.2026.1865303 (PMC13341934; doi:10.3389/fpsyt.2026.1865303)
Supplement: Supplementary file 1 [file Supplementaryfile1.docx]

**Supplemental Materials**

Table S1. The search strategies.

| Databases | Step | Search Strategies |
| --- | --- | --- |
| PubMed | #1 | "[neoplasms](https://www.ncbi.nlm.nih.gov/mesh/68009369)"[Mesh Terms] Sort by: Best match |
|  | #2 | "advanced cancer"[Title/Abstract] OR "advanced neoplasm"[Title/Abstract] OR "terminal cancer"[Title/Abstract] Sort by: Best match |
|  | #3 | "depression"[Mesh Terms] Sort by: Best match |
|  | #4 | "depression"[Title/Abstract] OR "depressive symptoms"[Title/Abstract] OR "depressive disorder"[Title/Abstract] OR "mood disorder"[Title/Abstract] Sort by: Best match |
|  | #5 | "palliative care"[Mesh Terms] Sort by: Best match |
|  | #6 | "palliative care"[Title/Abstract] OR "palliative medicine"[Title/Abstract] OR "end-of-life care"[Title/Abstract] OR "hospice care"[Title/Abstract] Sort by: Best match |
|  | #7 | "prevalence"[Mesh Terms] Sort by: Best match |
|  | #8 | "incidence"[Mesh Terms] Sort by: Best match |
|  | #9 | "e[pidemiology](https://www.ncbi.nlm.nih.gov/mesh/68004813)"[Mesh Terms] Sort by: Best match |
|  | #10 | "prevalence"[Title/Abstract] OR "incidence"[Title/Abstract] OR "e[pidemiology](https://www.ncbi.nlm.nih.gov/mesh/68004813)"[Title/Abstract] Sort by: Best match |
|  | #11 | #1 OR #2 Sort by: Best match |
|  | #12 | #3 OR #4 Sort by: Best match |
|  | #13 | #5 OR #6 Sort by: Best match |
|  | #14 | #7 OR #8 Sort by: Best match |
|  | #15 | #11 AND #12 AND #13 AND #14 Sort by: Best match |
| Web of Science | #1 | TS=([neoplasms](https://www.ncbi.nlm.nih.gov/mesh/68009369)) OR TS=(advanced cancer) OR TS=(advanced neoplasm) OR TS=(terminal cancer) |
|  | #2 | **Ts=(depression) OR Ts=(**depressive symptoms**) OR Ts=(**depressive disorder**) OR** TS=(mood disorder) |
|  | #3 | **Ts=(**palliative care**) OR Ts=(**palliative medicine**) OR Ts=(**end-of-life care**) OR** TS=(hospice care) |
|  | #4 | TS=(prevalence) OR **TS=(incidence) OR TS=(**e[pidemiology](https://www.ncbi.nlm.nih.gov/mesh/68004813)) |
|  | #5 | #1 AND #2 AND #3 AND #4 |
| Scopus | #1 | ABS("[neoplasms](https://www.ncbi.nlm.nih.gov/mesh/68009369)" OR "advanced cancer" OR "advanced neoplasm" OR "terminal cancer") |
|  | #2 | ABS("depression" OR "depressive symptoms" OR "depressive disorder" OR "mood disorder") |
|  | #3 | ABS ("palliative care" OR "palliative medicine" OR "end-of-life care" OR "hospice care") |
|  | #4 | ABS ("incidence" OR "prevalence" OR "e[pidemiology](https://www.ncbi.nlm.nih.gov/mesh/68004813)") |
|  | #5 | #1 AND #2 AND #3 AND #4 |
| Embase | **#1** | '[neoplasms](https://www.ncbi.nlm.nih.gov/mesh/68009369)'/exp |
|  | **#2** | 'advanced cancer':ti,ab,kw OR 'advanced neoplasm':ti,ab,kw OR 'terminal cancer':ti,ab,kw |
|  | **#3** | 'depression'/exp |
|  | **#4** | 'depression'**:ti,ab,kw OR** 'depressive symptoms'**:ti,ab,kw OR** 'depressive disorder'**:ti,ab,kw OR** 'mood disorder'**:ti,ab,kw** |
|  | **#5** | 'palliative care'/exp |
|  | **#6** | 'palliative care'**:ti,ab,kw OR** 'palliative medicine'**:ti,ab,kw OR** 'end-of-life care'**:ti,ab,kw OR** 'hospice care'**:ti,ab,kw** |
|  | **#7** | 'prevalence**':ti,ab,kw OR 'incidence':ti,ab,kw OR '**e[pidemiology](https://www.ncbi.nlm.nih.gov/mesh/68004813)**':ti,ab,kw** |
|  | **#8** | #1 OR #2 |
|  | **#9** | #3 OR #4 |
|  | **#10** | #5 OR #6 |
|  | **#11** | #7 AND #8 AND #9 AND #10 |
| Cochrane Library | #1 | **MeSH descriptor: [Neoplasms] explode all trees** |
|  | #2 | (advanced cancer):ti,ab,kw OR (advanced neoplasm):ti,ab,kw OR (terminal cancer):ti,ab,kw |
|  | #3 | **MeSH descriptor: [Depression] explode all trees** |
|  | #4 | **(**depressive symptoms**):ti,ab,kw OR (**depressive disorder**):ti,ab,kw OR (**mood disorder**):ti,ab,kw** |
|  | #5 | **MeSH descriptor: [P**alliative care**] explode all trees** |
|  | #6 | **(**palliative medicine**):ti,ab,kw OR (**end-of-life care**):ti,ab,kw OR (**hospice care**):ti,ab,kw** |
|  | #7 | (prevalence)**:ti,ab,kw OR (incidence**)**:ti,ab,kw OR (**e[pidemiology](https://www.ncbi.nlm.nih.gov/mesh/68004813))**:ti,ab,kw** |
|  | #8 | #1 OR #2 |
|  | #9 | #3 OR #4 |
|  | #10 | #5 OR #6 |
|  | #11 | #7 AND #8 AND #9 AND #10 |
| CINAHL | #1 | TI [neoplasms](https://www.ncbi.nlm.nih.gov/mesh/68009369) OR TI advanced cancer OR TI advanced neoplasm OR TI terminal cancer |
|  | #2 | **TI depression OR TI** depressive symptoms **OR TI** depressive disorder **OR** TI mood disorder |
|  | #3 | **TI** palliative care **OR TI** palliative medicine **OR TI** end-of-life care **OR** TI hospice care |
|  | #4 | TI prevalence OR **TI incidence OR TI** e[pidemiology](https://www.ncbi.nlm.nih.gov/mesh/68004813) |
|  | #5 | #1 AND #2 AND #3 AND #4 |
| PsycINFO | S1 | TI [neoplasms](https://www.ncbi.nlm.nih.gov/mesh/68009369) OR TI advanced cancer OR TI advanced neoplasm OR TI terminal cancer |
|  | S2 | **TI depression OR TI** depressive symptoms **OR TI** depressive disorder **OR** TI mood disorder |
|  | S3 | **TI** palliative care **OR TI** palliative medicine **OR TI** end-of-life care **OR** TI hospice care |
|  | S4 | TI prevalence OR **TI incidence OR TI** e[pidemiology](https://www.ncbi.nlm.nih.gov/mesh/68004813) |
|  | S5 | S1 AND S2 AND S3 AND S4 |

Table S2. Quality assessment results of included studies.

| Study | Q1 | Q2 | Q3 | Q4 | Q5 | Q6 | Q7 | Q8 | Q9 | Overall |
| --- | --- | --- | --- | --- | --- | --- | --- | --- | --- | --- |
| Alotaibi and Alsuhail. 2025 | Y | N | Y | Y | Y | Y | Y | Y | Y | L |
| Amano et al. (2024) | Y | N | Y | Y | Y | Y | Y | Y | Y | L |
| Atinafu et al. (2022) | N | N | U | Y | Y | Y | Y | Y | U | M |
| Bovero et al. (2019) | Y | N | U | Y | Y | Y | Y | Y | U | M |
| Bovero et al. (2021) | Y | N | U | Y | Y | Y | Y | Y | U | M |
| Bovero et al. (2023) | Y | N | U | Y | Y | Y | Y | Y | N | M |
| Bužgová et al. (2015) | Y | N | Y | Y | Y | Y | Y | Y | Y | L |
| Chan et al. (2012) | Y | N | U | Y | Y | Y | Y | Y | U | M |
| Delgado-Guay et al. (2009) | Y | N | U | Y | Y | Y | Y | Y | U | M |
| Gontijo Garcia et al. (2023) | Y | N | U | Y | Y | Y | Y | Y | U | M |
| Grotmol et al. (2017) | Y | N | Y | Y | Y | Y | Y | Y | U | M |
| Islam et al. (2022) | Y | N | U | Y | Y | Y | Y | Y | Y | L |
| Jung et al. (2025) | Y | N | U | Y | Y | Y | Y | Y | N | M |
| Lloyd-Williams et al. (2014) | Y | N | Y | Y | Y | Y | Y | Y | U | M |
| Mercadante et al. (2015) | Y | N | Y | Y | Y | Y | Y | Y | U | M |
| Mercadante et al. (2017) | Y | N | Y | Y | Y | Y | Y | Y | N | M |
| Mercadante et al. (2021) | Y | N | Y | Y | Y | Y | Y | Y | N | M |
| Mystakidou et al. (2009) | Y | N | U | Y | Y | Y | Y | Y | Y | L |
| Mystakidou et al. (2012) | Y | N | U | Y | Y | Y | Y | Y | Y | L |
| O'Connor et al. (2010) | Y | N | Y | Y | Y | Y | Y | Y | U | M |
| Park et al. (2018) | Y | N | U | Y | Y | Y | Y | Y | Y | L |
| Pérez-Cruz et al. (2019) | Y | N | U | Y | Y | Y | Y | Y | U | M |
| Rojas-Concha et al. (2023) | Y | N | U | Y | Y | Y | Y | Y | U | M |
| Sela. (2007) | Y | N | U | Y | Y | Y | Y | Y | Y | L |
| Sewtz et al. (2021) | Y | N | U | Y | Y | Y | Y | Y | Y | L |
| Slovacek et al. (2009) | Y | N | U | Y | Y | Y | Y | Y | Y | L |
| Smith et al. (2003) | Y | N | U | Y | Y | Y | Y | Y | Y | L |
| Sudarisan et al. (2019) | Y | N | U | Y | Y | Y | Y | Y | U | M |
| Truong et al. (2024) | Y | N | U | Y | Y | Y | Y | Y | Y | L |

Notes: Y: Yes, N: No, U: Unclear. L: Low risk, M: Moderate risk, H: High risk.

Q1: Was the sample frame appropriate to address the target population?

Q2: Were study participants sampled in an appropriate way?

Q3: Was the sample size adequate?

Q4: Were the study subjects and the setting described in detail?

Q5: Was the data analysis conducted with sufficient coverage of the identified sample?

Q6: Were valid methods used for the identification of the condition?

Q7: Was the condition measured in a standard, reliable way for all participants?

Q8: Was there appropriate statistical analysis?

Q9: Was the response rate adequate, and if not, was the low response rate managed appropriately?


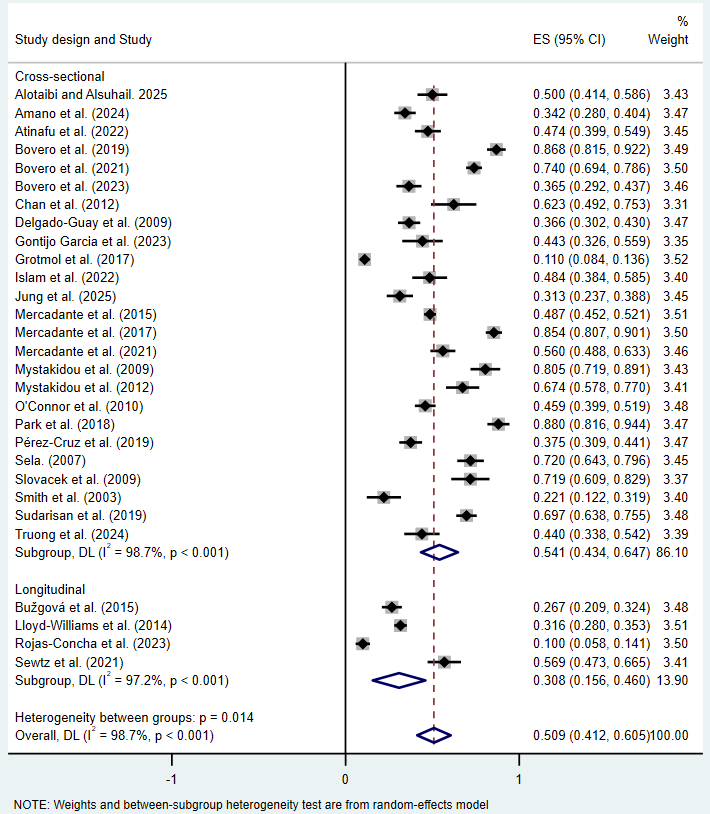


Figure S1. The pooled prevalence of depression in patients with advanced cancer receiving palliative care based on study design.


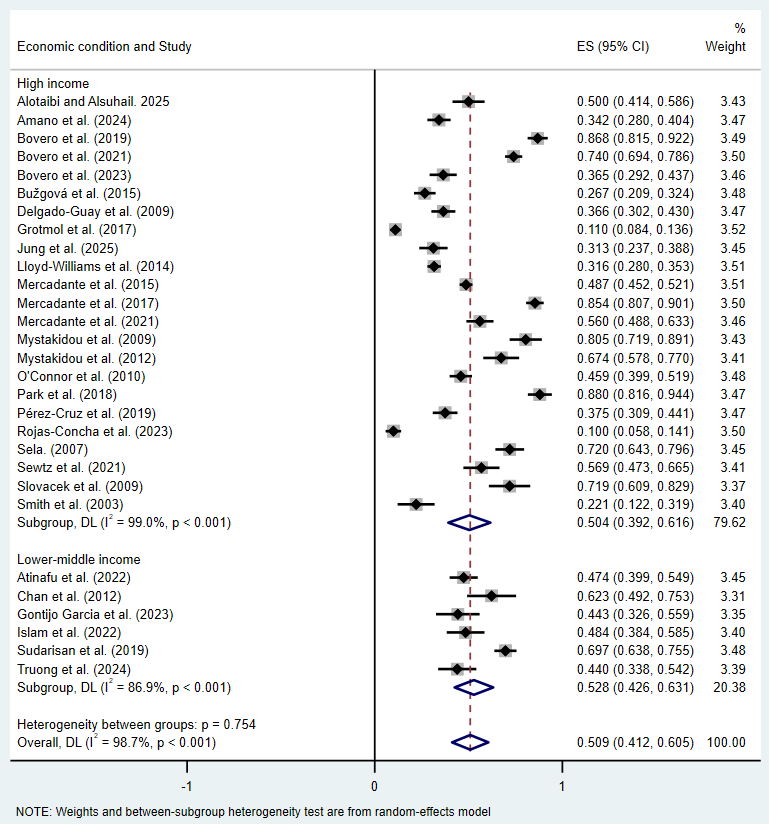


Figure S2. The pooled prevalence of depression in patients with advanced cancer receiving palliative care based on economic condition.


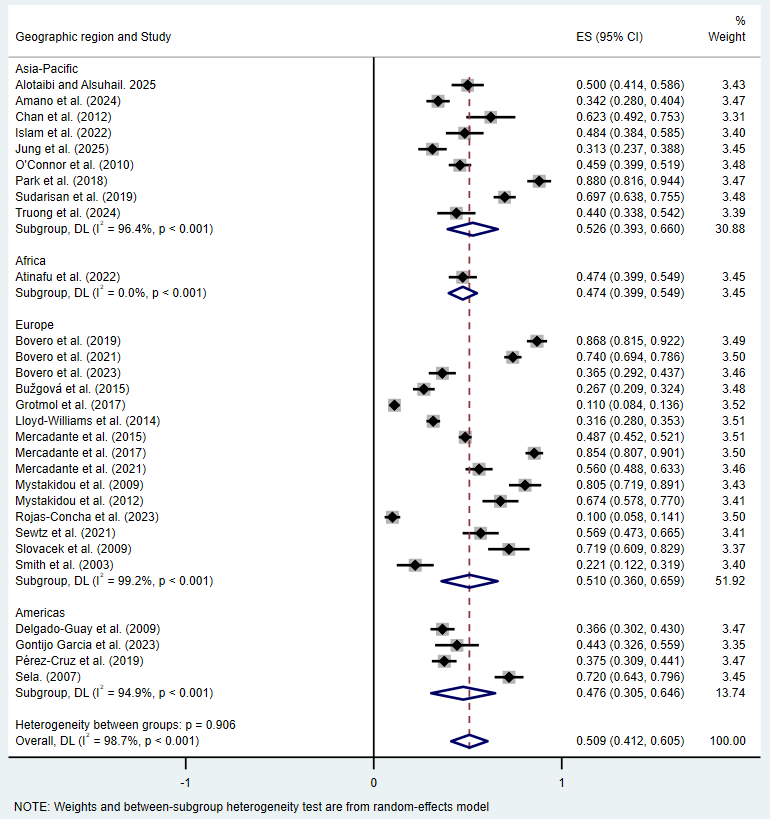


Figure S3. The pooled prevalence of depression in patients with advanced cancer receiving palliative care based on geographic region.


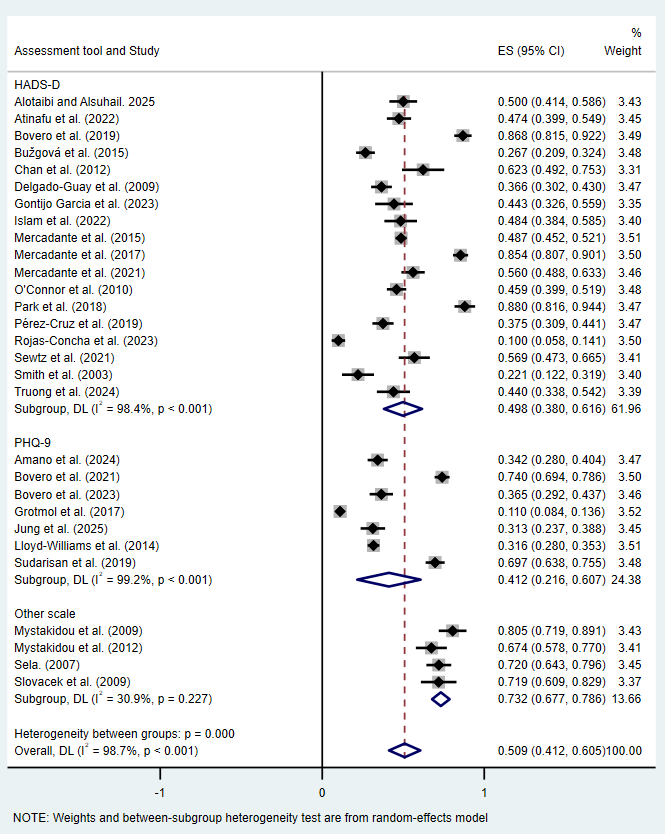


Figure S4. The pooled prevalence of depression in patients with advanced cancer receiving palliative care based on assessment tool and cut-off.


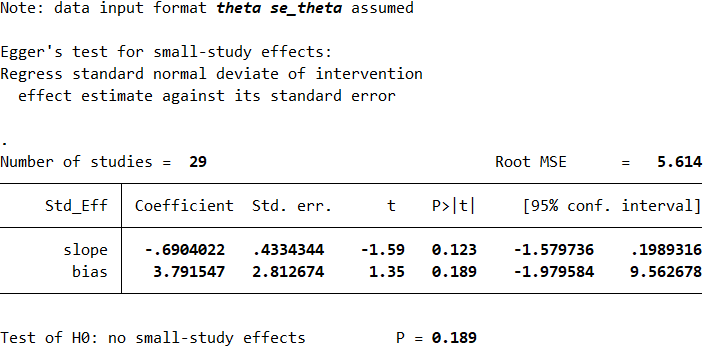


Figure S5. Egger's test results.

Table S3. The results of the sensitivity analysis.

| **Omitted Study** | **Prevalence** | **95% CI** |
| --- | --- | --- |
| Alotaibi and Alsuhail. 2025 | 0.509 | (0.426, 0.592) |
| Amano et al. (2024) | 0.515 | (0.432, 0.597) |
| Atinafu et al. (2022) | 0.510 | (0.426, 0.593) |
| Bovero et al. (2019) | 0.495 | (0.416, 0.575) |
| Bovero et al. (2021) | 0.500 | (0.418, 0.582) |
| Bovero et al. (2023) | 0.514 | (0.431, 0.596) |
| Bužgová et al. (2015) | 0.517 | (0.436, 0.599) |
| Chan et al. (2012) | 0.505 | (0.422, 0.588) |
| Delgado-Guay et al. (2009) | 0.514 | (0.431, 0.596) |
| Gontijo Garcia et al. (2023) | 0.511 | (0.428, 0.594) |
| Grotmol et al. (2017) | 0.523 | (0.445, 0.601) |
| Islam et al. (2022) | 0.509 | (0.426, 0.593) |
| Jung et al. (2025) | 0.516 | (0.433, 0.598) |
| Lloyd-Williams et al. (2014) | 0.516 | (0.433, 0.598) |
| Mercadante et al. (2015) | 0.509 | (0.426, 0.593) |
| Mercadante et al. (2017) | 0.496 | (0.416, 0.575) |
| Mercadante et al. (2021) | 0.507 | (0.423, 0.590) |
| Mystakidou et al. (2009) | 0.498 | (0.417, 0.579) |
| Mystakidou et al. (2012) | 0.503 | (0.420, 0.585) |
| O'Connor et al. (2010) | 0.510 | (0.427, 0.594) |
| Park et al. (2018) | 0.495 | (0.416, 0.574) |
| Pérez-Cruz et al. (2019) | 0.513 | (0.431, 0.596) |
| Rojas-Concha et al. (2023) | 0.523 | (0.446, 0.601) |
| Sela. (2007) | 0.501 | (0.419, 0.583) |
| Sewtz et al. (2021) | 0.506 | (0.423, 0.590) |
| Slovacek et al. (2009) | 0.501 | (0.419, 0.583) |
| Smith et al. (2003) | 0.519 | (0.438, 0.599) |
| Sudarisan et al. (2019) | 0.502 | (0.419, 0.584) |
| Truong et al. (2024) | 0.511 | (0.428, 0.594) |
